# Droj2 Facilitates Somatosensory Neurite Sculpting via GTP-Binding Protein Arf102F in *Drosophila*

**DOI:** 10.3390/ijms241713213

**Published:** 2023-08-25

**Authors:** Menglong Rui, Weiyu Kong, Wanting Wang, Ting Zheng, Su Wang, Wei Xie

**Affiliations:** 1 The Key Laboratory of Developmental Genes and Human Disease, School of Life Science and Technology, Southeast University, Nanjing 210096, China; 2 Co-Innovation Center of Neuroregeneration, Nantong University, Nantong 226019, China

**Keywords:** *Drosophila*, neuroscience, development, remodeling

## Abstract

Developmental remodeling of neurite is crucial for the accurate wiring of neural circuits in the developing nervous system in both vertebrates and invertebrates, and may also contribute to the pathogenesis of neuropsychiatric disorders, for instance, autism, Alzheimer’s disease (AD), and schizophrenia. However, the molecular underpinnings underlying developmental remodeling are still not fully understood. Here, we have identified DnaJ-like-2 (Droj2), orthologous to human DNAJA1 and DNAJA4 that is predicted to be involved in protein refolding, as a developmental signal promoting dendrite sculpting of the class IV dendritic arborization (C4da) sensory neuron in *Drosophila*. We further show that Arf102F, a GTP-binding protein previously implicated in protein trafficking, serves downstream of Droj2 to govern neurite pruning of C4da sensory neurons. Intriguingly, our data consistently demonstrate that both Droj2 and Arf102F promote the downregulation of the conserved L1-type cell-adhesion molecule Neuroglian anterior to dendrite pruning. Mechanistically, Droj2 genetically interacts with Arf102F and promotes Neuroglian downregulation to initiate dendrite severing. Taken together, this systematic study sheds light on an unprecedented function of Droj2 and Arf102F in neuronal development.

## 1. Introduction

During animal development, a vital step in the establishment and maintain functional neural circuits in the brain is selectively eliminating superfluous neuronal connections without loss of the parental neurons, referred to as pruning [1]. Neuronal pruning is an evolutionarily conserved process broadly occurring in both vertebrates and invertebrates during the development of the nervous systems and is crucial for sculpting the late nervous system [2,3,4,5]. Research over the past few years has illustrated that in vertebrates, neurons in the central nervous system (CNS), including the hippocampus, cortex, cerebellum, and olfactory bulb would prune their inappropriate neuronal connections [1,6]. While, in invertebrates, like the holometabolous insects such as *Drosophila*, neurons of the larval nervous system undergo stereotyped remodeling of axons and dendrites in the central and peripheral nervous systems during early metamorphosis [4,7]. In the CNS, bifurcated axons of larval mushroom body (MB) γ neurons remove their dorsal and medial axon bundles as well as the whole dendrites and subsequently regrow the medial part to form the novel adult-specific areas [8,9,10]. In the peripheral nervous system (PNS), Class IV dorsal dendritic arborization neurons, also known as C4da sensory neurons of *Drosophila*, selectively remove their larval dendrites to refine the nervous system during metamorphosis [11]. Of note, the underlying mechanisms of neuronal remodeling during *Drosophila* metamorphosis share superficial similarities with Wallerian neuronal degeneration and neurodegenerative diseases [12,13,14]. In the past few decades, several lines of evidence have suggested that abnormalities of neuronal pruning are associated with neuropsychiatric disorders, including autism, schizophrenia, and Alzheimer’s disease (AD) [15,16]. Thus, understanding the molecular mechanisms of neuronal pruning will provide an important insight into neurite fragmentation and elimination during development in neuropsychiatric disorders.

Dendrite pruning is governed in a cell-intrinsic manner and initiates in response to a late larval pulse of the steroid hormone ecdysone. A heterodimer complex, consisting of ecdysone receptor B1 (EcR-B1) and Ultraspiracle, works together with two epigenetic factors, Brahma and CREB-binding protein, to induce the downstream transcriptional cascade after binding to ecdysone [8,11,17,18]. Subsequently, Sox14 and the Headcase are triggered and activate the expression of the actin disassembly factor Mical [19]. Several known factors, signal pathways, and physiological processes have also been identified in the regulation of dendrite pruning in *Drosophila*, including ubiquitin-proteasomal degradation [10,11,20], caspases activity [21,22], compartmentalized calcium transients [23,24], microtubule disassembly [25,26,27,28], microtubule orientation [14,29,30], JNK signaling [31], energy metabolism [32], and mechanical tearing [33].

It is worth noting that the dendrite severing of somatosensory neurons is triggered through both global and local endocytosis by way of Rab5/ESCRT-dependent endolysosomal degradation. It has been well documented that the conserved L1-type cell-adhesion molecule Neuroglian is internalized from the plasma membrane into early endosomes and undergoes endolysosomal degradation to trigger the onset of dendrite severing [34]. Previous studies have shown that Sec71 functions as a GEF for the small GTPase Arf1 to regulate the dendrite pruning of *Drosophila* sensory neurons [35]. Yif1 interacts with Yip1 on Golgi and promotes dendrite pruning in sensory neurons during the metamorphosis of *Drosophila* [36]. The membrane protein Raw controls dendrite pruning via the secretory pathway [37]. All of these signaling pathways facilitate sensory neurite severing via the secretory pathway in the manner of endolysosomal degradation of Neuroglian. After severing, the residual dendrites are rapidly fragmented directed by local caspase activity [22], after which the epidermal cells have been reported as the primary phagocytes responsible for the clearance of the fragmented dendrites [38]. Despite the tremendous advance toward the understanding of dendrite remodeling during the past years, the molecular and cellular basis underlying neuronal sculpting is still under investigation.

To systematically understand the mechanisms of neurite remodeling, we took advantage of an unbiased RNA interference (RNAi) screen to identify new candidates for governing dendrite pruning. Herein, we report that DnaJ-like-2 (Droj2), orthologous to human DNAJA1 and DNAJA4, is required for dendrite pruning of the C4da neuron. A previous study implicated the crucial role of Droj2 in NF-κB signaling [39]. Moreover, it has been documented that Droj2 is predicted to be involved in protein refolding [40]. Interestingly, in this study, we identify that Droj2 is involved in the regulation of dendrite remodeling through Arf102F. Arf102F is a GTP-binding protein, initially predicted as a regulator of vesicle budding and uncoating within the Golgi apparatus [40]. Previous analysis has uncovered that Arf102F deficiency in border cells disrupts the integrity of the Golgi structure. As a consequence, Golgi is redistributed evenly in the cluster, and only a few Golgi puncta are visible after ablating Arf102F [41,42]. Our data consistently elucidate the important role of Droj2 and Arf102F in Golgi distribution. We further demonstrate that Droj2 and Arf102F maintain the membrane localization of the conserved L1-type cell-adhesion molecule Neuroglian, and this event is essential for initiating dendrite severing. Thus, our data present a cell-autonomous role of Droj2 and Arf102F in neurite remodeling that has not yet been documented in both vertebrates and invertebrates.

## 2. Results

### 2.1. Droj2 Is Required for Neurite Remodeling of C4da Sensory Neuron

**Figure 1:**
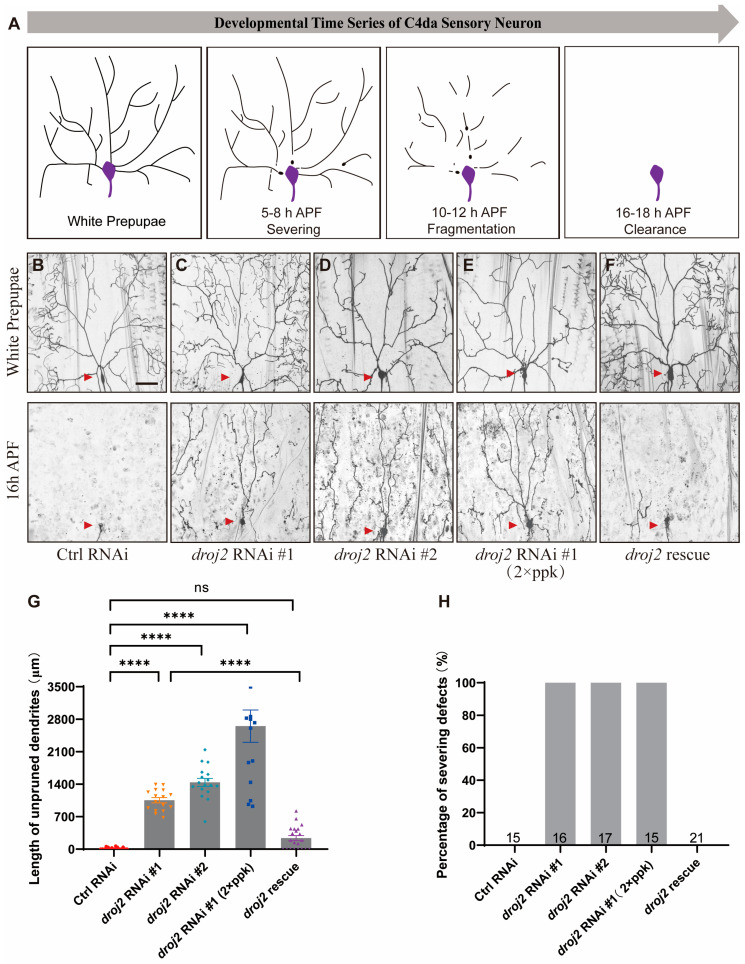
Ablating Droj2 Causes Dendritic Sculpting Defects in C4da Sensory Neuron. (**A**) A schematic of dendritic elimination in C4da sensory neurons during the development of *Drosophila*. (**B**–**F**) Live confocal images of C4da neurons expressing UAS–CD4–tdGFP driven by ppk–Gal4 at the WP and 16 h APF stages. Dendrites of control RNAi (**B**), *droj2* RNAi #1 (**C**), *droj2* RNAi #2 (**D**), *droj2* RNAi #1 with two copies of ppk–Gal4 (**E**), and *droj2* rescue (**F**) C4da neurons at the WP and 16 h APF stages. Red arrowheads point to the somas of C4da sensory neurons. (**G**,**H**) Quantitative analysis of uncleared dendritic length and percentage of severing defects at 16 h APF. The number of neurons (n) examined in each group is shown as dots on each bar, and the genetypes are distinguished with different colors. In G, data are mean ± s.e.m. One-way ANOVA with Bonferroni’s test was applied to determine statistical significance. ns, not significant; **** *p* < 0.0001. The number of neurons (n) examined in each group is shown on the bars. Scale bar: 50 μm.

Previous studies have unambiguously implied that both progressive and regressive events are indispensable for the establishment of mature nervous systems during an animal’s development. A variety of neurons remodel by eliminating excessive or incorrect larval connections subsequent to the regrowth of adult-specific processes during metamorphosis in *Drosophila*. It has been well-documented that there are types of C4da neurons, including ddaC, v’ada, and vdaB, ranging from dorsal to ventral in each hemi-segment of *Drosophila* larvae. Analogical dendrite pruning manner appeared in all C4da neurons, whereas the pruning time of v’ada and vdaB neurons is marginally delayed during metamorphosis. Moreover, vdaB neurons are usually lost at the adult stage [11]. Thus, ddaC neurons, termed C4da neurons in the following text, are extensively utilized for the phenomenological delineation of dendrite remodeling [31,37]. Invariably, the primary and secondary dendritic branches of C4da neurons are severed from the soma 5–6 h after puparium formation (APF), and this process is recognized as the initial stage of pruning. Subsequently, severed neurites are ablated via degeneration and epithelial phagocytosis [38,43]. Ultimately, dendrites are cleared, while somas and axons remain intact at 16–18 h APF [11] (Figure 1A).

To systematically obtain insights into the molecular mechanisms that underlie neurite pruning, we conducted an unbiased RNAi screen, making use of a class IV da sensory neuron driver, pickpocket-Gal4 (ppk-Gal4), to attenuate gene function in the C4da neuron. From this screen, we isolated two independent RNAi lines, TH02998.N (#1) and BL57382 (#2), that were delineated by a prominent deficiency in dendrite pruning. Both the RNAi lines target the gene of *droj2*, a molecular chaperone homology with human DNAJA1 and DNAJA4, and the RNAi knockdown of *droj2* consistently led to a pronounced deficit in dendrite pruning in C4da neurons at 16 h APF (Figure 1C,D,G,H). In contrast, dendrites were completely ablated in the control neurons at the same time point (Figure 1B,G,H). Likewise, knockdown with other *droj2* RNAi lines, V104880 (#3) and V23637 (#4), also resulted in similar pruning defects at 16 h APF (Figure S1A–E). Moreover, the expression of the *droj2* RNAi transgene (#1) with two copies of ppk-Gal4 drivers caused more severe deficiencies with a residual with an average dendritic length of 2650 μm at 16 h APF (Figure 1E,G,H). In an attempt to further shed light on the crucial role of Droj2 in neurite pruning, we next constructed a transgene using the UASt promoter for the high-level expression of Droj2 in C4da neurons. Throughout our experiments, we observed that the residual dendrite branches in *droj2* RNAi neurons were substantially eliminated via the reintroduction of UAS-Droj2 at 16 h APF (Figure 1F–H). Additionally, we examined the transcription level of *droj2* via RT-PCR (Reverse Transcription-Polymerase Chain Reaction), and UAS-Droj2 overexpression, to some extent, enhances the gene expression of *droj2* in Droj2 RNAi (Figure S2). In summary, these results systematically indicate that Droj2 plays a novel and pivotal role in the regulation of dendrite remodeling of *Drosophila* somatosensory neurons.

### 2.2. Loss of Arf102F Function Leads to Dendrite Remodeling Defects in C4da Neuron

**Figure 2:**
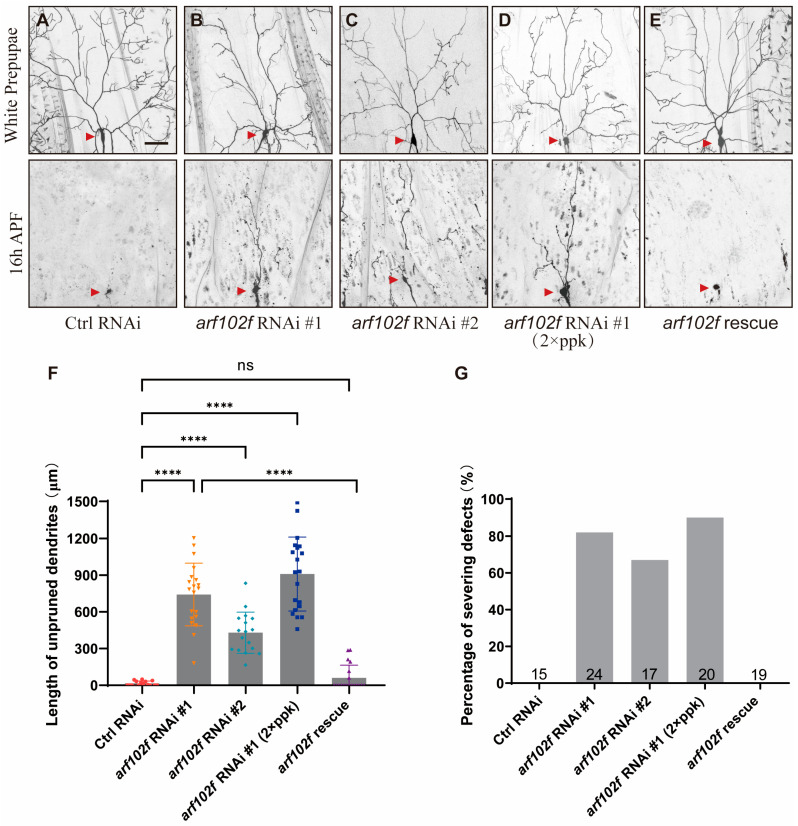
Arf102F is Required for Dendrite Remodeling in C4da Sensory Neuron. (**A**–**E**) Live confocal images of C4da neurons expressing UAS–CD4–tdTomato by ppk–Gal4 at the WP stage or 16 h APF. Neurons expressing *arf102f* RNAi #1 (**B**), *arf102f* RNAi #2 (**C**), *arf102f* RNAi #1 with two copies of ppk–Gal4 (**D**), and *arf102f* rescue (**E**) showed consistent dendrite pruning defects at 16 h APF, as compared to the wild-type neurons (**A**). Red arrowheads point to the C4da somas. (**F**,**G**) Quantitative analysis of uncleared dendritic length and percentage of severing defects at 16 h APF. The number of neurons (n) examined in each group is shown as dots on each bar, and the genetypes are distinguished with different colors. In F, data are mean ± s.e.m. One-way ANOVA with Bonferroni’s test was applied to determine statistical significance. ns, not significant; **** *p* < 0.0001. The number of neurons (n) examined in each group is shown on the bars. Scale bar: 50 μm.

As Droj2 is predicted to be involved in protein-refolding, it is likely that this process involves downstream-regulated proteins that are also required for the dendrite pruning of *Drosophila* C4da sensory neurons. In order to identify the downstream target of Droj2 in neurite remodeling, we performed another round of RNAi screening with our collection of RNAi lines based on the genes that might be correlated with Droj2 according to the FlyBase database. Eventually, we isolated another two independent RNAi lines, BL27268 (#1) and V12931 (#2), which displayed dendrite pruning deficiencies. Both RNAi lines correspond to the same gene, *arf102f*, which encodes a GTP-binding protein involved in protein trafficking. The known functions of Arf102F include the intracellular regulation of vesicle budding and uncoating within the Golgi apparatus and sustaining the integrity of the Golgi structure [40,41,42]. Unexpectedly, database searches also implied that Droj2 might govern protein processing, including Arf102F [44]. We thus asked whether Arf102F is also involved in the regulation of the dendrite pruning of C4da neurons. RNAi knockdown of *arf102f* via ppk-Gal4 was found to lead to deficits in dendrite pruning at 16 h APF. By contrast, all larval dendrites were completely removed in the control RNAi neurons at this time point (control-0%, RNAi #1–82%, and RNAi #2–67%; Figure 2A–C,F,G). In order to confirm this result, we used another independent RNAi, THU2594 (#3), and a p-element insertion allele, *arf102f*^c339^, and our data showed that these mutant clones exhibited consistent dendrite pruning defects, whereas wild-type neurons completely pruned their larval dendrites at 16 h APF (Figure S3A–E). To further ascertain the requirement of Arf102F in dendrite pruning, we then integrated UAS-*arf102f* RNAi with ppk-Gal4 in the same fly and expressed double copies of UAS-*arf102f* RNAi with double copies of ppk driver. As a consequence, the length of uncleared dendrites and the percentages of severing defects in C4da neurons were further enhanced to approximately 909 μm and 90%, respectively. (Figure 2D,F,G). To verify that the deficit in dendrite pruning resulting from the expression of UAS-*arf102f* RNAi was not an off-target effect, we conducted a rescue experiment. We attempted to alleviate the phenotypic defects by overexpressing UAS-Arf102F. Unambiguously, UAS-Arf102F expressed in C4da neurons substantially ameliorated the dendrite pruning deficiencies against a background of Arf102F knockdown. Indeed, none of the C4da neurons had attached dendrites and only an average 61 μm dendritic length residual at 16h APF when UAS-*arf102f* RNAi was co-expressed with UAS-Arf102F (Figure 2E–G). Taken together, these results collectively underscore the importance of Arf102F in the neurite remodeling of C4da neurons.

### 2.3. Droj2 Genetically Interacts with Arf102F to Promote Neurite Elimination

**Figure 3:**
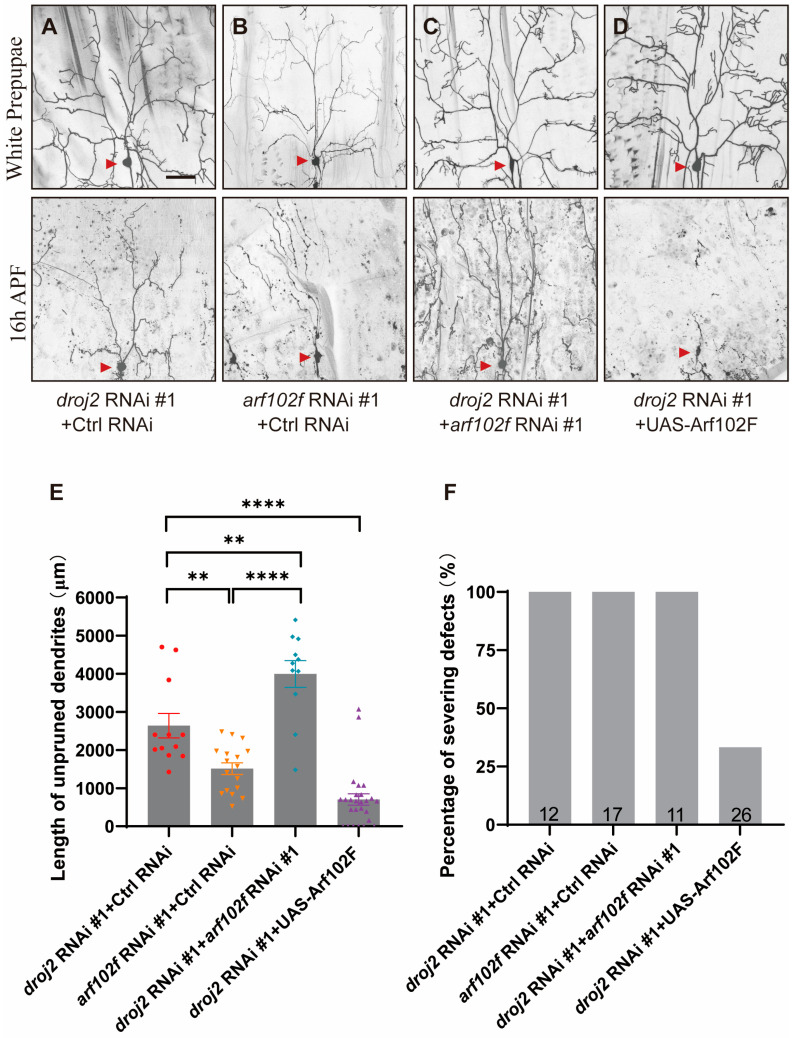
Droj2 and Arf102F Genetically Interact During Dendritic Pruning in C4da Sensory Neuron. (**A**–**D**) Live confocal images of C4da neurons visualized by ppk–Gal4–driven UAS–CD4–tdGFP expression at the WP stage or 16 h APF. Simultaneous knockdown of Droj2 and Arf102f via co-expressing *droj2* RNAi and *arf102f* RNAi (**C**) in C4da neurons enhanced the dendrite pruning deficiency, compared to those neurons co-expressing control RNAi + *droj2* RNAi (**A**) and control RNAi + *arf102f* RNAi (**B**). Reintroduction of UAS–Arf102F expression in C4da neurons substantially rescued the dendrite pruning deficiency in *droj2* knockdown background (**D**). Red arrowheads point to the C4da somas. (**E**,**F**) Quantitative analysis of unpruned dendrite lengths and percentages of C4da neurons showing severing defects at 16 h APF. The number of neurons (n) examined in each group is shown as dots on each bar, and the genetypes are distinguished with different colors. In E, data are mean ± s.e.m. One-way ANOVA with Bonferroni’s test was applied to determine statistical significance. ** *p* < 0.01; **** *p* < 0.0001. The number of neurons (n) examined in each group is shown on the bars. Scale bar: 50 μm.

As Droj2 was implied to govern the protein processing of Arf102F, we proposed that Droj2 might act together with Arf102F to facilitate the dendrite pruning of C4da neurons during the development of *Drosophila*. In order to ascertain whether Droj2 and Arf102F are components of the same genetic pathway during neurite pruning, we co-expressed RNAi lines in C4da neurons to cause the double knockdown of Droj2 and Arf102F and compared the results with their single-RNAi phenotypes. We found that *droj2* RNAi knockdown with the control gene resulted in more prominent dendrite branches with residual C4da neurons than *arf102f* RNAi plus control RNAi at 16 h APF (Figure 3A,B,E,F). Furthermore, a significant increase was also found in neurite pruning deficiencies in C4da neurons co-expressing both *droj2* and *arf102f* RNAi transgenes, in contrast with *droj2* or *arf102f* RNAi plus control gene knockdown (Figure 3A–C,E,F). It is conceivable that Arf102F works downstream of Droj2 in neurite remodeling. Thus, we subsequently overexpressed Arf102F in Droj2-ablated sensory neurons, and our data suggested that the remodeling defects in dendrite in *droj2* RNAi were rescued (Figure 3A,D–F), indicating that Droj2 developmentally facilitates the dendrite elimination of sensory neurons via Arf102F. To confirm the proposed epistasis of genetic interaction, we next overexpressed Droj2 in *arf102f* RNAi, and the result suggested that Droj2 induction is unable to alleviate the dendrite remodeling defect in Arf102F downregulated neurons (Figure S4A–C). To further examine the potential interaction between Droj2 and Arf102F, we decided to determine whether the knockdown of Droj2 could influence the protein levels or localization of Arf102F. To this end, we generated an antibody against Arf102F but were unable to probe the endogenous protein in our immunostaining assay. Therefore, we ordered transgenes expressing Arf102F fused with a green fluorescent protein (GFP) and HA tag at their C terminus (UAS-Arf102F-GFP and UAS-Arf102F-HA) to visualize the subcellular distribution of Arf102F. Importantly, the expression of Arf102F-HA and Arf102F-GFP in C4da neurons completely restored the deficits in neurite pruning in *arf102f* RNAi neurons (UAS-Arf102F-HA-Figure 2E–G; UAS-Arf102F-GFP-unpublished data), illustrating that these UAS transgenes could functionally substitute endogenous Arf102F. In addition, we combined control RNAi and *droj2* RNAi with ppk-Gal4 and subsequently took advantage of these lines to drive the expression of Arf102F-HA. Our immunostaining and statistical results showed that, in comparison with the control, the attenuation of Droj2 led to a prominent decline in Arf102F-HA intensity (Figure S5A–C), further validating the regulatory relationship between Droj2 and Arf102F. Collectively, our data systematically elucidate that Droj2 and Arf102F act in a common pathway to govern neurite remodeling during the metamorphosis of *Drosophila*.

### 2.4. Arf102F Localizes on Golgi Compartments and Regulates the Integrity of Golgi Apparatus in C4da Neuron

**Figure 4:**
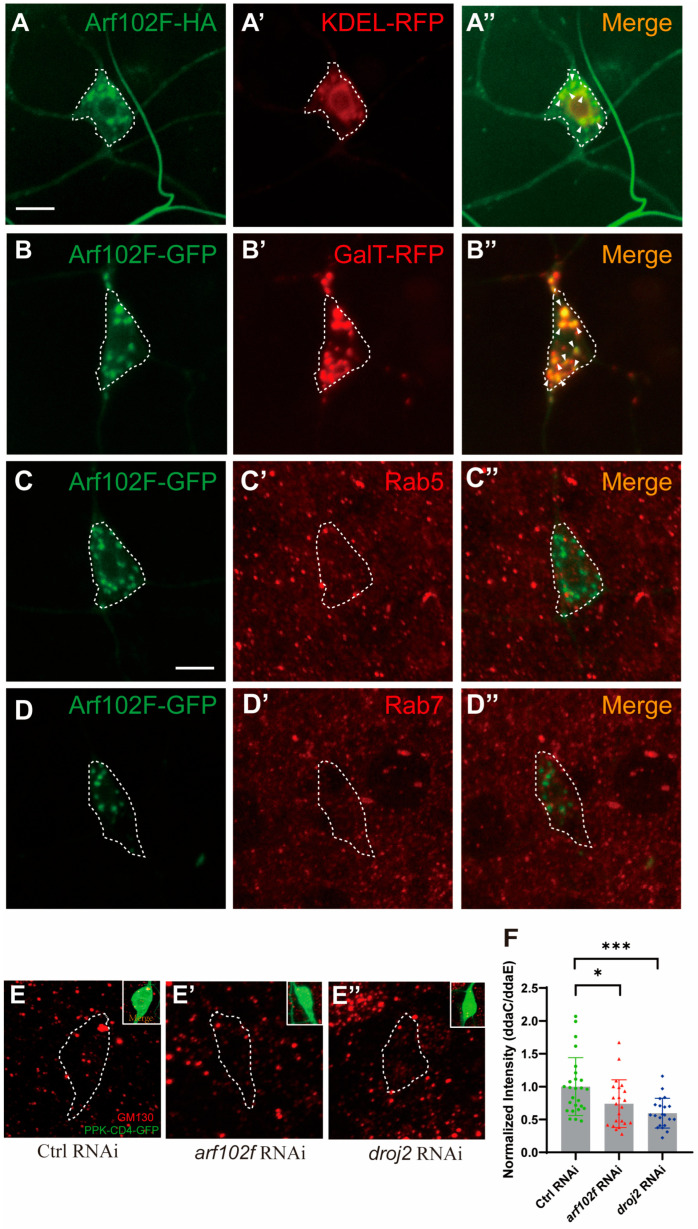
Arf102F Localizes on ER/Golgi Compartments and Regulates the Integrity of Golgi Apparatus in C4da Neuron. (**A**–**D**) Confocal images of C4da neurons that were labelled for Arf102f–HA and KDEL-RFP (**A**–**A’’**), Arf102f-GFP and GalT–RFP (**B**–**B’’**), Arf102f–GFP and Rab5 (**C**–**C’’**), Arf102f–GFP and Rab7 (**D**–**D’’**) at the late WP stage. White arrowheads indicate co-localized portions. (**E**–**E’’**) Confocal images of Ctrl RNAi (**E**), *arf102f* RNAi (**E’**), *droj2* RNAi (**E’’**) C4da neurons expressing UAS–CD4–tdGFP driven by ppk–Gal4 that were labelled for GM130. C4da somas are outlined with dashed lines. (**F**) Quantitative analysis of normalized GM130 fluorescence intensities in C4da somas. The number of neurons (n) examined in each group is shown as dots on each bar, and the genetypes are distinguished with different colors. In (**F**), data are mean ± s.e.m. One-way ANOVA with Bonferroni’s test was applied to determine statistical significance. * *p* < 0.05; *** *p* < 0.001. The number of neurons (n) examined in each group is shown on the bars. Scale bars: 10 μm.

A previous study has illustrated that Arf102F is involved in the regulation of vesicle budding and uncoating within the Golgi apparatus [40]. To characterize the subcellular localization of Arf102F, we then attempted to investigate whether Arf102F localizes on the ER or Golgi, serving as a constituent to sustain their integrity. To this end, we expressed Arf102F-HA and Arf102F-GFP under the control of the ppk-Gal4 driver to assay the distribution, and Arf102F-HA and Arf102F-GFP were shown to contain numerous discrete punctate structures (Figure 4A–D). To further gain insight into the identity of these punctate structures, we co-expressed Arf102F-HA with the ER marker (KDEL-RFP) and Arf102F-GFP with the Golgi marker (GalT-RFP). We observed that Arf102F was co-localized, to some extent, with the ER apparatus (Figure 4A–A’’). However, a more striking co-localization was revealed between Arf102F and Golgi-RFP-positive puncta (Figure 4B–B’’). Given that both the secretory and endocytosis pathways are required to control neurite clearance during the metamorphosis of *Drosophila*, we next sought to determine whether Arf102F is also involved in the Rab5/ESCRT-mediated endolysosomal process. However, the Arf102F-HA punctate pattern did not overlap with those of the early endosomal marker Rab5 (Figure 4C–C’’) or the late endosomal marker Rab7 (Figure 4D–D’’). Therefore, our findings indicate that Arf102F is predominantly localized on Golgi and partly on ER.

A previous analysis of Arf102F distribution prompted us to speculate on the potential function of Arf102F in the regulation of the Golgi apparatus’ integrity during dendrite pruning. To evaluate whether Arf102F is required for the proper localization of Golgi marker, we carried out an immunostaining experiment with the antibody against GM130, an endogenous cis-Golgi marker, and found that GM130 was localized as numerous bright, discrete punctate structures in the control soma. Intriguingly, these GM130 puncta signals were diminished after Arf102F was knocked down in C4da neurons (Figure 4E,E’,F). Likewise, the GM130 punctate intensity was also reduced in *droj2* RNAi neurons, in contrast to the bright puncta in the control neurons (Figure 4E,E’’,F). Hence, our results imply that Droj2 and Arf102F play a vital role in governing the integrity of Golgi compartments and might be involved in the regulation of the intracellular protein secretion process.

### 2.5. Droj2 and Arf102F Are Indispensable for the Distribution of Neuroglian

In an attempt to assay the molecular underpinnings for the neurite pruning deficiency resulting from the functionally compromised Droj2 and Arf102F, we first wondered whether Droj2 and Arf102F knockdown influences ecdysone-associated gene expression. However, the ecdysone target genes *headcase* and *ecr-b1* were normally expressed in C4da neurons at the onset of the pupal phase upon Droj2 and Arf102F knockdown (unpublished data). The secretory pathway has been well-documented to facilitate the endolysosomal degradation of Neuroglian [35,36,37]. An important prior study suggested that Rab5/ESCRT-dependent endocytosis and lysosomal degradation of the L1-type cell-adhesion molecule Neuroglian is required for the promotion of dendrite pruning during early metamorphosis. In control, C4da neurons, Neuroglian is strikingly redistributed to the early endosome at 6 h APF, the initial dendrite severing point, and subsequently, its protein level is extremely declined by lysosomal degradation [34,45].

**Figure 5:**
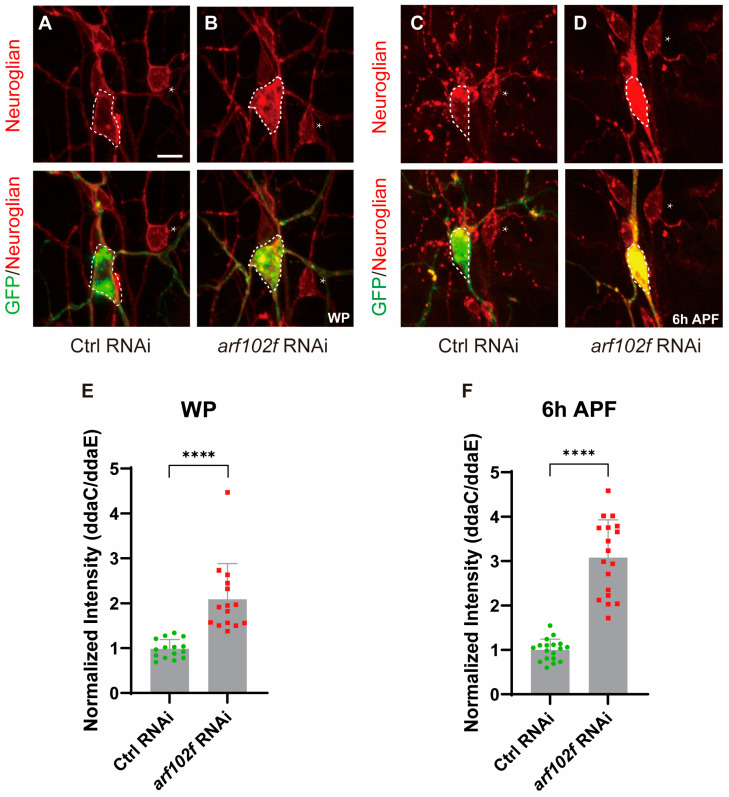
Arf102F is Required for the Downregulation of the Cell-adhesion Molecule Neuroglian. (**A**,**B**) Confocal images of control RNAi (**A**) and *arf102f* RNAi (**B**) C4da neurons that were immunostained for Neuroglian at the WP stage. (**C**,**D**) Confocal images of control RNAi (**C**) and *arf102f* RNAi (**D**) C4da neurons that were immunostained for Neuroglian at 6 h APF stage. ddaC somas are outlined with dashed lines, ddaE somas are indicated with asterisks. (**E**,**F**) Quantitative analysis of normalized Neuroglian fluorescence intensities of the indicated genotypes in C4da somas at the WP (**E**) and 6 h APF (**F**) stages. The number of neurons (n) examined in each group is shown as dots on each bar, and the genetypes are distinguished with different colors. In (**E**,**F**), data are mean ± s.e.m. Two-tailed Student’s *t*-test was applied to determine statistical significance. **** *p* < 0.0001. Scale bars: 10 μm.

**Figure 6:**
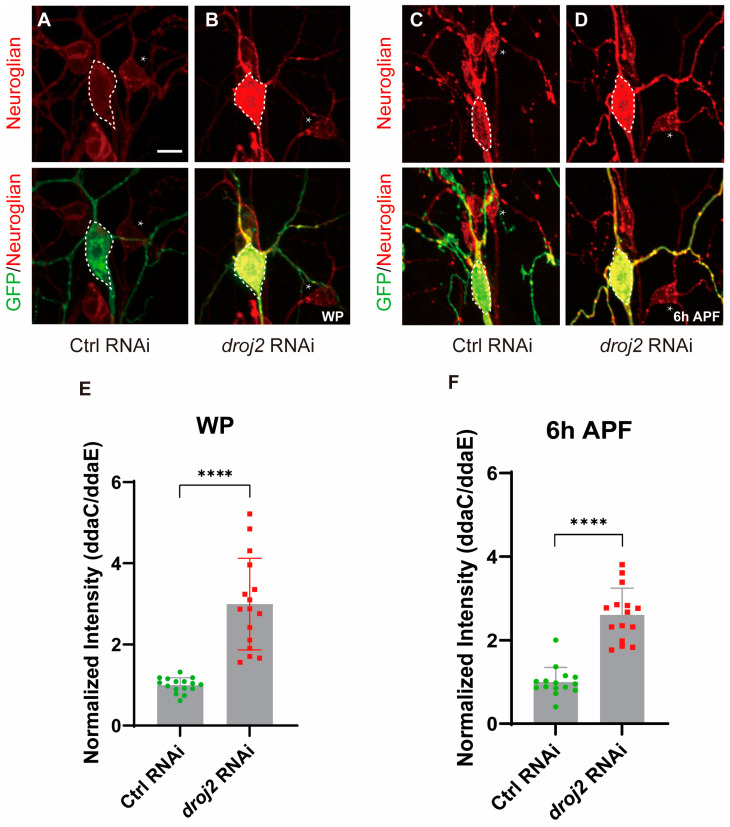
Droj2 is Required for Downregulation of the Cell-adhesion Molecule Neuroglian. (**A**,**B**) Confocal images of control RNAi (**A**) and *droj2* RNAi (**B**) C4da neurons that were immunostained for Neuroglian at the WP stage. (**C**,**D**) Confocal images of control RNAi (**C**) and *droj2* RNAi (**D**) C4da neurons that were immunostained for Neuroglian at 6 h APF stage. ddaC somas are outlined with dashed lines, ddaE somas are indicated with asterisks. (**E**,**F**) Quantitative analysis of normalized Neuroglian fluorescence intensities of the indicated genotypes in C4da somas at the WP (**E**) and 6 h APF (**F**) stages. The number of neurons (n) examined in each group is shown as dots on each bar, and the genetypes are distinguished with different colors. In (**E**,**F**), data are mean ± s.e.m. Two-tailed Student’s *t*-test was applied to determine statistical significance. **** *p* < 0.0001. Scale bars: 10 μm.

As Arf102F has been suggested to control the vesicle budding and uncoating of the Golgi apparatus, we, therefore, wanted to know whether Droj2 and Arf102F are involved in the protein redistribution of Neuroglian. Firstly, in an attempt to determine Neuroglian distribution in Arf102F-attenuated neurons, we expressed RNAi via ppk-Gal4 to knock down Arf102F in C4da neurons. Intriguingly, we observed a 2.1-fold enhancement in Neuroglian levels, as detected by the BP104 antibody, when compared to the wild-type in the WP phase (Figure 5A,B,E). More strikingly, the protein level of Neuroglian was increased by over threefold in *arf102f* RNAi neurons at 6 h APF (Figure 5C,D,F). Next, we interrogated whether Droj2 also contributes to maintaining normal Neuroglian levels in C4da neurons during the development of *Drosophila*. At the WP stage, we isolated Droj2, which, when compromised by RNAi, led to a drastic augmentation of Neuroglian intensity in C4da neurons in contrast to controls (Figure 6A,B,E). To further verify the role of Droj2 in Neuroglian regulation, we also investigated the distribution of Neuroglian at 6 h APF, and our data showed that the ablation of Droj2 resulted in a remarkable accumulation of Neuroglian in the soma of C4da neurons (Figure 6C,D,F). Subsequently, we asked whether Arf102F works downstream of Droj2 to regulate Neuroglian levels. We overexpressed Arf102F in *droj2* RNAi and Droj2 in *arf102f* RNAi neurons at the WP stage, respectively. Our results showed that Arf102F overexpression can partially rescue the Nrg defect in Droj2 downregulated neurons, whereas induction of Droj2 is unable to ameliorate the Nrg defect in Arf102F downregulated neurons (Figure S6A–D). These data collectively reveal the indispensable role of Droj2 and Arf102F in the developmental downregulation of the neuronal membrane protein Neuroglian at the onset of the pupal stage.

### 2.6. Droj2 and Arf102F Regulate Neurite Pruning by Downregulating Neuroglian

**Figure 7:**
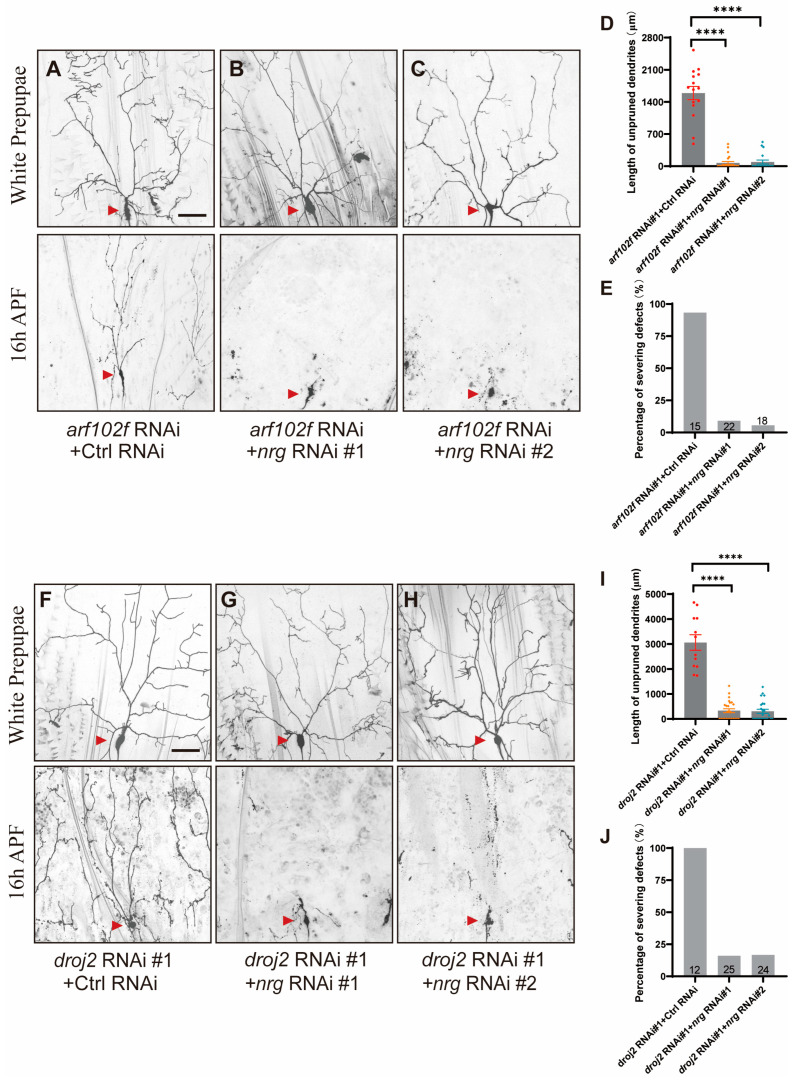
Droj2 and Arf102F Govern Neurite Remodeling via Downregulation of Neuroglian. (**A**–**C**) Live confocal images of C4da neurons expressing UAS–CD4–tdGFP driven by ppk–Gal4 at the WP and 16 h APF stages. Dendrites of *arf102f* RNAi + Control RNAi (**A**), *arf102f* RNAi + *neuroglian* RNAi #1 (**B**) and *arf102f* RNAi + *neuroglian* RNAi #2 (**C**) C4da sensory neurons. (**F**–**H**) Live confocal images of C4da neurons expressing UAS–CD4–tdGFP driven by ppk–Gal4 at the WP and 16 h APF stages. Dendrites of *droj2* RNAi + Control RNAi (**F**), *droj2* RNAi + *neuroglian* RNAi #1 (**G**) and *droj2* RNAi + *neuroglian* RNAi #2 (**H**) C4da sensory neurons. Red arrowheads point to the C4da somas. (**D**,**E**,**I**,**J**) Quantitative analysis of unpruned dendrite lengths and percentages of C4da neurons showing severing defects at 16 h APF. The number of neurons (n) examined in each group is shown as dots on each bar, and the genetypes are distinguished with different colors. In (**D**,**I**), data are mean ± s.e.m. One-way ANOVA with Bonferroni’s test was applied to determine statistical significance. **** *p* < 0.0001. The number of neurons (n) examined in each group is shown on the bars. Scale bar: 50 μm.

In order to ascertain whether Droj2 and Arf102F’s promotion of dendrite pruning can indeed be attributed to aberrant Neuroglian accumulation, we first knocked down Neuroglian, via two independent *neuroglian* RNAi transgenes, BL38215 (#1) and BL37496 (#2), in Arf102F RNAi-expressing C4da sensory neurons. Notably, the expression of two independent *neuroglian* RNAi transgenes, which has been shown to efficiently knock down Neuroglian [35,37], drastically rescued the neurite pruning phenotype in *arf102f* RNAi C4da sensory neurons (Figure 7A–E), supporting the idea that, analogous to Rab5, Arf1/Sec71, and Raw [34,35,37], Arf102F contributes to the endocytosis and degradation of Neuroglian to facilitate neurite pruning. Analogously, the knockdown of Neuroglian, via these two RNAi lines, also significantly suppressed the neurite pruning deficiency in Droj2-compromised neurons (Figure 7F–J). Overall, our data uncover an unprecedented function of Droj2 and Arf102F: developmentally facilitating the downregulation of Neuroglian prior to the neurite pruning of C4da sensory neurons.

## 3. Discussion

During animal development, precise neural circuit assembly is achieved through heterogeneous tightly controlled events including neurogenesis, neural differentiation, formation of new axons and synaptic connections, and the elimination of exuberant and erroneous neurons and synapses [1,3]. Neuronal remodeling is an evolutionarily conserved process and is critical to the appropriate development of both vertebrate and invertebrate nervous systems. Perturbations in neuronal remodeling have been considered a potential etiology of autism, AD, and schizophrenia [46,47,48]. How neuronal remodeling is precisely governed, is a fundamental biological question that has not been fully resolved.

Previous works have implied that the GTP-binding protein Arf102F is a regulator of vesicle budding and uncoating within the Golgi apparatus involved in protein trafficking [40,49]. In addition, Droj2, orthologous to human DNAJA1 and DNAJA4, has been predicted to be a heat shock protein family Hsp40 member involved in protein refolding, including Arf102F [40]. However, the important functions of Droj2 and Arf102F in neuronal development have never been examined. In this study, we provide multiple lines of in vivo evidence to demonstrate that Droj2 and Arf102F are novel regulators of the neurite pruning of *Drosophila* somatosensory neurons. Genetic assays with divergence in RNAi transgenes, together with the rescue results, consistently reveal that Droj2 and Arf102F are essential for the neurite pruning of *Drosophila* C4da neurons. Given the fact that dendrite sculpting defeat in Arf102F is less severe than in Droj2, and the phenotype in Droj2-compromised neurons cannot be completely restored by Arf102F overexpression, it raises the possibility that signaling, including other family members of ADP-ribosylation factor (ARF), downstream of Droj2, might also be involved in the regulation of somatosensory neurite remodeling. Therefore, further investigation is required to elucidate the unknown downstream factors that could contribute to the demonstration of a detailed regulatory mechanism of neurite sculpting by Droj2. Additionally, we also found a decreased Arf102F signal in Droj2 downregulated neurons. We propose that Droj2 is involved in the regulation of the protein processing and maturation of Arf102. The downregulation of Droj2 might cause erroneous refolding and modifications to Arf102 that could facilitate protein degradation. Future investigations are warranted to elucidate a detailed regulatory mechanism regarding the effect of Droj2 on Arf102F.

How does Droj2/Arf102F facilitate the neurite pruning of C4da neurons during the metamorphosis of *Drosophila*? In order to study the underlying regulatory mechanism, we next performed experiments to monitor the subcellular localization of Arf102F. It is notable that the discrete punctate signals of Arf102F-GFP were co-localized, to some extent, with the ER apparatus, and conspicuously co-localized with a Golgi marker. We next examined the distribution of the Golgi marker in Droj2 and Arf102F mutant neurons, and our data showed that the loss of Droj2 and Arf102F function resulted in a prominent decrease in Golgi marker intensity, suggesting that Arf102F might function in Golgi as an auxiliary regulator to modulate protein secretion. Previously, it has been well-documented that Neuroglian, an evolutionarily conservative L1-type cell adhesive molecule, is internalized from the plasma membrane to early endosomes via Rab5/ESCRT-dependent endocytosis and profoundly degraded by the endolysosomal pathway to activate neurite severing [34]. The secretory pathway has been reported to contribute to abnormal Neuroglian membrane distribution, endolysosomal degradation, and ultimately neurite pruning of *Drosophila* C4da neurons [35,36,37]. Intriguingly, we observed that the attenuation of Droj2 and Arf102F consistently led to the remarkable accumulation of Neuroglian in C4da neurons, indicating that Droj2 and Arf102F facilitate the degradation of Neuroglian prior to neurite pruning. How does Droj2/Arf102F impair Neuroglian distribution? It is plausible that an ambiguous ligand could be governed by Arf102F and be specifically secreted via the secretory pathway to trigger Neuroglian endocytosis, and hence promote the clearance of fractured dendritic branches of C4da neurons during the metamorphosis of *Drosophila*. Thus, in future studies, it would be interesting to search for the ligand or secreted protein that is regulated by Droj2 and Arf102F to trigger Neuroglian endocytosis and degradation. Of note, the human ortholog of Droj2, DnaJA1 has been shown to bind physically with Abeta, facilitating the aggregation of Abeta peptides, and downregulation of this chaperone protects against Abeta-mediated toxicity in AD models [50,51]. Additionally, in a previous study it was found that overexpression of Arf102F can reverse spine degeneration in primary neurons from an AD-related apolipoprotein (APO) E4 mouse model [52]. Intriguingly, excessive spine production or inappropriate pruning may take place in autism, increasing the number of spines. Whereas in AD, spines are rapidly pruned or lost in late adulthood, resulting in a reduction in the number of spines and weakened cognitive function [16]. Therefore, understanding the molecular underpinnings of Droj2 and Arf102F in neurite remodeling may provide insight into the etiologies of AD and autism and might reveal novel drug targets.

## 4. Materials and Methods

### 4.1. Fly Strains

The following stocks were obtained from Bloomington Stock Centre (BSC, Bloomington, IN, USA): mCherry RNAi (BL35785), *droj2* RNAi #2 (BL57382), *arf102f* RNAi #1 (BL27268), *arf102f*^c339^ (BL16341), UAS-Arf102f-GFP (BL65866), UAS-Arf102f-HA (BL93855), *neuroglian* RNAi #1 (BL38215), *neuroglian* RNAi #2 (BL37496), UAS-GalT-RFP (BL30902), UAS-KDEL-RFP (BL30909). The following stocks were obtained from the Vienna *Drosophila* RNAi Centre (VDRC): *droj2* RNAi #3 (V104880), *droj2* RNAi #4 (V23637), *arf102f* RNAi #2 (V12931). The following stocks were obtained from the Tsinghua RNAi Stock Center: *droj2* RNAi #1 (TH02998.N), *arf102f* RNAi #3 (THU2594). All the flies were raised at 25 °C on standard medium (corn flour: 50 g/L; sugar: 37 g/L; yeast: 24.5 g/L; agar: 8 g/L; propionic acid: 4 mL/L; antibiotic: 1.75 g/L).

### 4.2. Generation of Droj2 Transgene

For the rescue experiment, a UAS-Droj2-HA construct was made by cloning the full-length cDNA into the transformation vector pUAST-attB. The transgenic fly of UAS-Droj2-HA was generated by standard germline transformation. cDNA of Droj2 was obtained from reverse transcription of genomic mRNA. Fly head mRNA was extracted using TRIzol (Life Technologies, Carlsbad, CA, USA), and reverse transcribed using cDNA Reverse Transcriptase Kit (Applied Biosystems, Waltham, MA, USA).

### 4.3. Live Imaging Analysis

*Drosophila* prepupae at the WP stage were first washed in PBS buffer briefly for three times and subsequently immersed with 90% glycerol. For imaging C4da neurons, pupal cases at 16 h APF were carefully removed antecedent to mount with 90% glycerol. Images of C4da neurites were captured with Zeiss LSM 900 confocal microscope.

### 4.4. Immunohistochemistry

For immunostaining, larvae and pupae were dissected in pre-cooling PBS and fixed with 4% formaldehyde for 30 min. The experimental and control samples were incubated synergistically in the same tubes. Mounting was conducted in the mounting medium of VectaShield, and the samples were directly visualized by a Zeiss LSM 900 confocal microscope. The following primary antibodies were used for immunohistochemistry at the indicated dilution: mouse anti-Neuroglian (1:25, BP104, DSHB, Iowa City, IA, USA), rabbit anti-GM130 (1:200, ab52649, Abcam, Cambridge, UK), rabbit anti-HA (1:500, C29F4, Cell Signaling, Danvers, MA, USA), mouse anti-Rab5 (1:250, 610281, BD Transduction Laboratories, San Diego, CA, USA), mouse anti-Rab7 (1:20, DSHB). The following secondary antibodies were used for immunohistochemistry at the indicated dilution: Alexa Fluor 488- and Alexa Fluor 555- conjugated secondary antibodies (1:500, Invitrogen, Waltham, MA, USA).

### 4.5. Quantification of Dendrites

The structure of the C4da neuron dendrite at the WP and 16 h APF stages were captured via live confocal imaging. The length of unpruned dendrites and percentage of severing defects are utilized to evaluate the neurite remodeling phenotype. The length of unpruned dendrites was measured in a 275 µm × 275 µm region derived from the dorsal dendritic field of C4da neurons, ranging from the abdominal segments 2–4. The percentage of dendritic severing defects was defined by the percentage of neurons with larval dendrites attached to the soma at 16 h APF.

### 4.6. RNAi Screen

The unbiased RNAi screen is based on the stocks collected in our lab for identifying synaptic function regulators, which contain about 200 lines. Among these, we isolated Droj2, which plays a vital function in neurite remodeling. To further identify the downstream targets of Droj2 in neurite remodeling, we next searched the database at http://flybase.org/reports/FBgn0038145#interactions (accessed on 2 September 2021) (Click “Interactions” at the right sidebar of the page), from which it shows the genes that physically interact with Droj2. Subsequently, we ordered RNAi lines of these candidate genes to perform the second-round screening. Among these, we found that Arf102F is also required for neurite remodeling, and our data further confirmed that Arf102F works downstream of Droj2 in neurite remodeling.

### 4.7. Statistical Analysis

For pairwise comparison, a two-tailed Student’s *t*-test was used to determine statistical significance. For multiple-group comparison, one-way ANOVA with Bonferroni’s test was used to determine significance. The error bars in all graphs represent s.e.m. Statistical significance was defined as **** *p* < 0.0001, *** *p* < 0.001, ** *p* < 0.01, * *p* < 0.05, and ns, not significant. The presented data are from three independent experiments and the number of neurons (n) in each group is shown on the bars.

## Data Availability

Not applicable.

## Supplementary Materials

The supporting information can be downloaded at: https://www.mdpi.com/article/10.3390/ijms241713213/s1.
