# Supplementary material for: Droj2 Facilitates Somatosensory Neurite Sculpting via GTP-Binding Protein Arf102F in Drosophila
Source: Int J Mol Sci. 2023 Aug 25;24(17):13213. doi: 10.3390/ijms241713213 (PMC10487878; doi:10.3390/ijms241713213)
Supplement: Supplementary file 1 [file ijms-24-13213-s001.zip › ijms-2528401-supplementary.pdf]

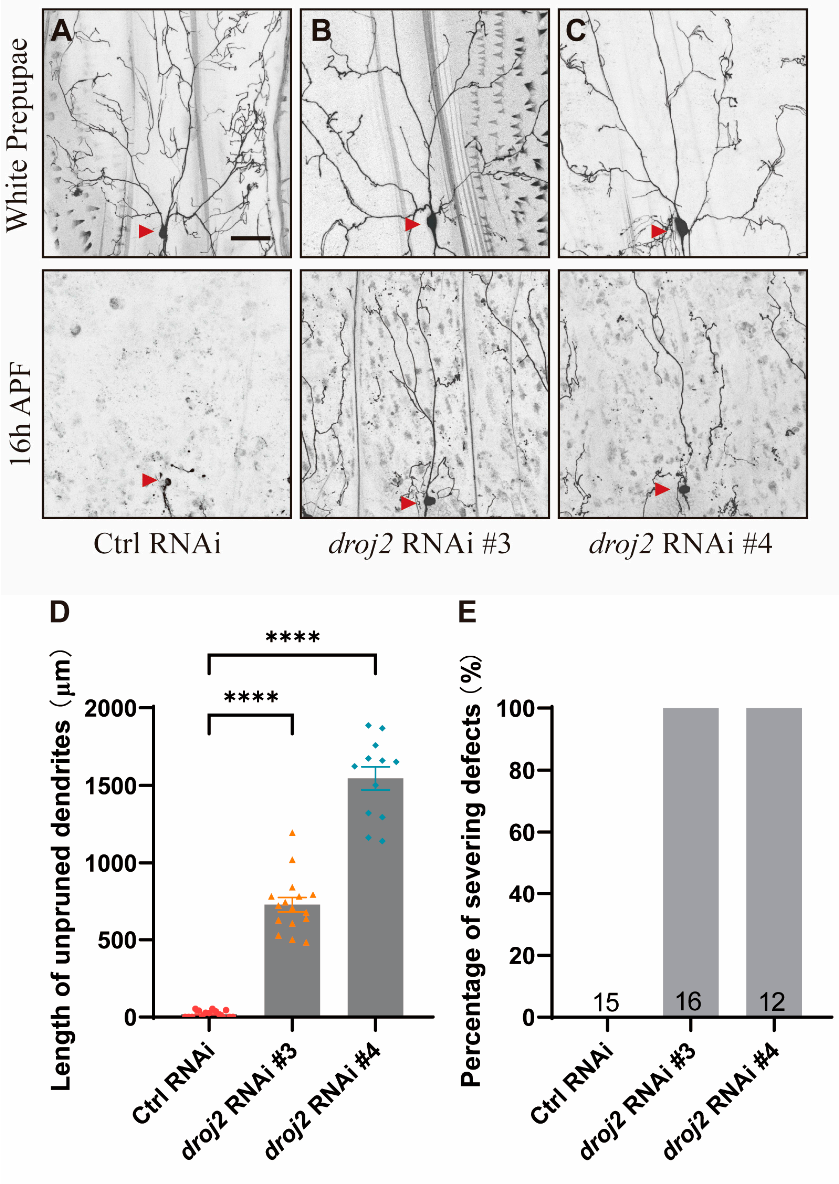

Figure S1. Droj2 is Essential for Dendritic Pruning of C4da Sensory Neuron. (A–C) Live confocal images of C4da neurons expressing UAS–CD4–tdGFP by ppk–Gal4 at the WP stage or 16 h APF. Neurons expressing *droj2* RNAi #3 (B) and *droj2* RNAi #4 (C) showed consistent dendrite pruning defects at 16 h APF, as compared to the control neurons (A). Red arrowheads point to the C4da somas. (D-E) Quantitative analysis of uncleared dendritic length and percentage of severing defects at 16 h APF. The number of neurons (n) examined in each group is shown as dots on each bar, and the genetypes are distinguished with different colors. In D, data are mean±s.e.m. One-way ANOVA with Bonferroni's test was applied to determine statistical significance. ns, not significant; ****$P<0.0001$. The number of neurons (n) examined in each group is shown on the bars. Scale bar: 50 μm.

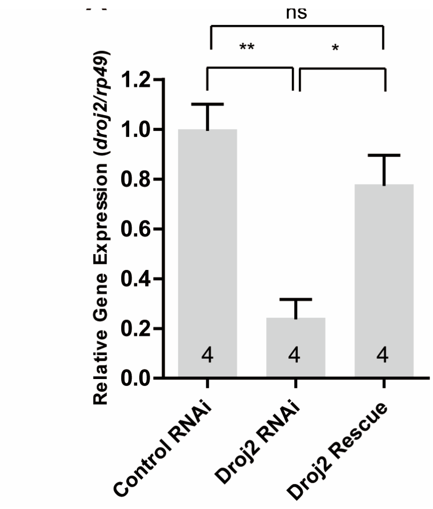

Figure S2. Droj2 Overexpression, to Some Extent, Enhances the Gene Expression of *droj2* in Droj2 RNAi. RT-PCR analysis and quantification of the relative mRNA level for control RNAi, *droj2* RNAi + UAS–Control, and *droj2* RNAi + UAS–Droj2, showing the mRNA level of *droj2* is increased after overexpressing Droj2 in Droj2 RNAi. Data are mean±s.e.m. One-way ANOVA with Bonferroni's test was applied to determine statistical significance. ns, not significant; *P<0.05; **P<0.01. The number of replicates (n) examined in each group is shown on the bars, 10 flies for each genotype per replicate.

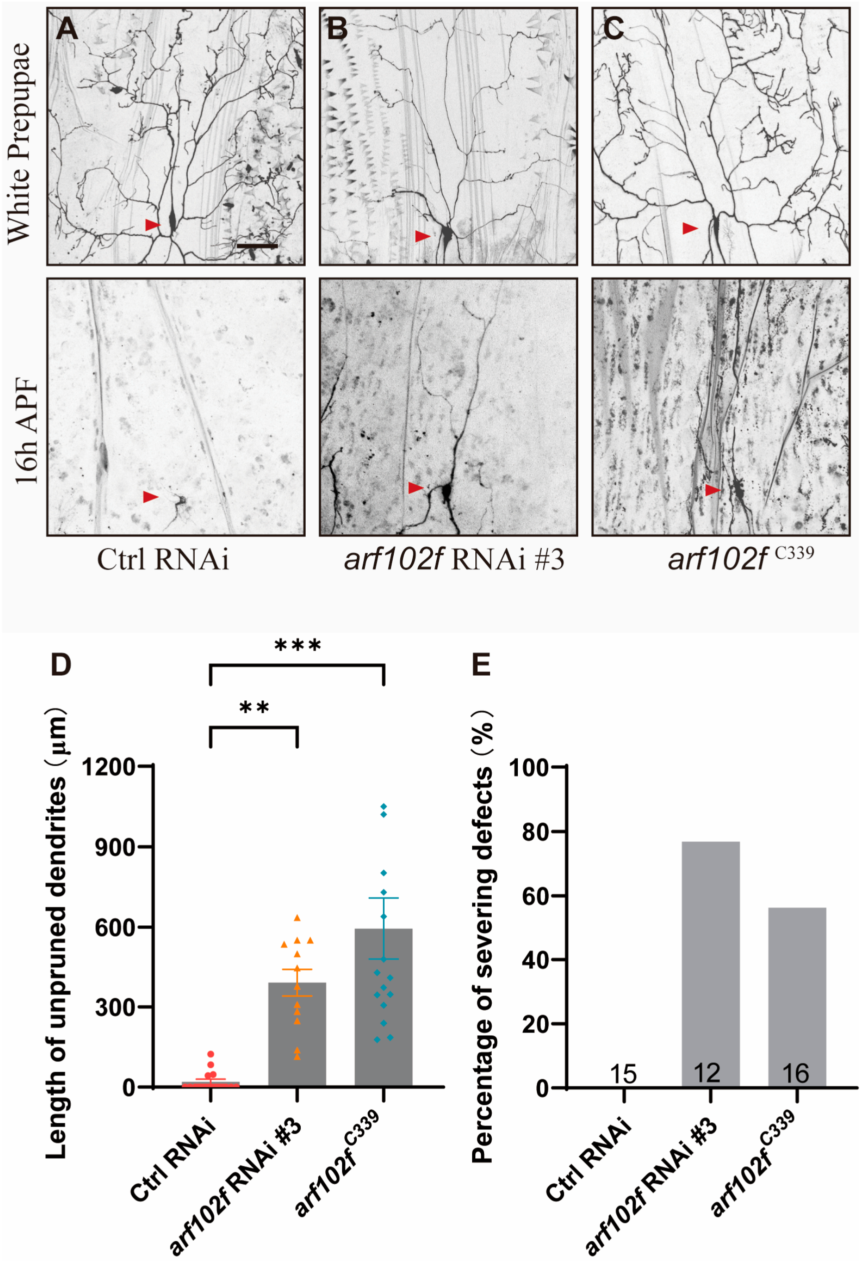

Figure S3. Arf102F is Essential for Dendritic Pruning of C4da Sensory Neuron. (A–C) Live confocal images of C4da neurons expressing UAS–CD4–tdTomato driven by ppk–Gal4 at the WP and 16 h APF stages. Dendrites of control RNAi (A), *arf102f* RNAi #1 (B), and a p-element insertion allele, *arf102f*^{c339} (C) C4da neurons at the WP and 16 h APF stages. Red arrowheads point to the somas of C4da sensory neurons. (D–E) Quantitative analysis of uncleared dendritic length and percentage of severing defects at 16 h APF. The number of neurons (n) examined in each group is shown as dots on each bar, and the genetypes are distinguished with different colors. In D, data are mean±s.e.m. One-way ANOVA with Bonferroni's test was applied to determine statistical significance. ns, not significant; **P<0.01; ***P<0.001. The number of neurons (n) examined in each group is shown on the bars. Scale bar: 50 μm.

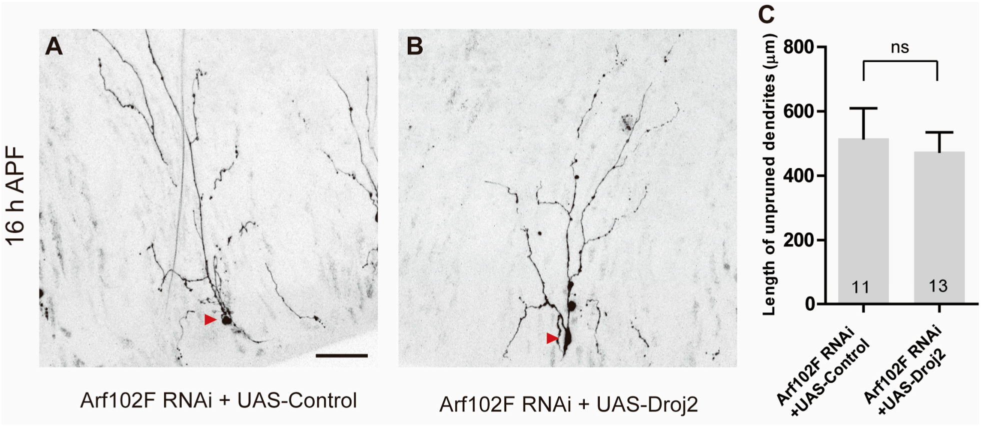

Figure S4. Droj2 Overexpression is Unable to Rescue the Dendrite Pruning Defect in Arf102F Downregulated Neurons. (A–B) Live confocal images of C4da neurons expressing UAS–CD4–tdTomato driven by ppk–Gal4 at 16 h APF stage. Dendrites of *arf102*f RNAi + UAS–Control (A) and *arf102*f RNAi + UAS–Droj2 (B). Red arrowheads point to the somas of C4da sensory neurons. (C) Quantitative analysis of uncleared dendritic length at 16 h APF. In C, data are mean±s.e.m. Two-tailed Student's t-test was applied to determine statistical significance. ns, not significant. The number of neurons (n) examined in each group is shown on the bars. Scale bar: 50 µm.

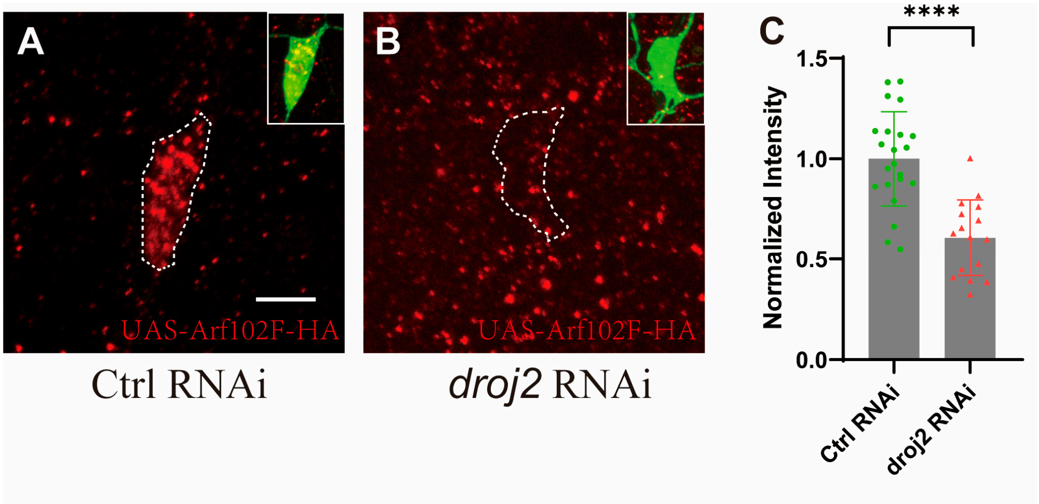

Figure S5. Loss of Droj2 Leads to Decline of Arf102F Intensity. (A–B) Confocal images of Ctrl RNAi (A) and *droj2* RNAi (B) C4da neurons expressing UAS–CD4–tdGFP driven by ppk–Gal4 that were labelled for Arf102f–HA. C4da somas are outlined with dashed lines. (C) Quantitative analysis of normalized fluorescence intensities of Arf102f–HA in C4da somas. The number of neurons (n) examined in each group is shown as dots on each bar, and the genetypes are distinguished with different colors. In C, data are mean±s.e.m. Two-tailed Student's t-test was applied to determine statistical significance. ****P<0.0001. Scale bars: 10 µm.

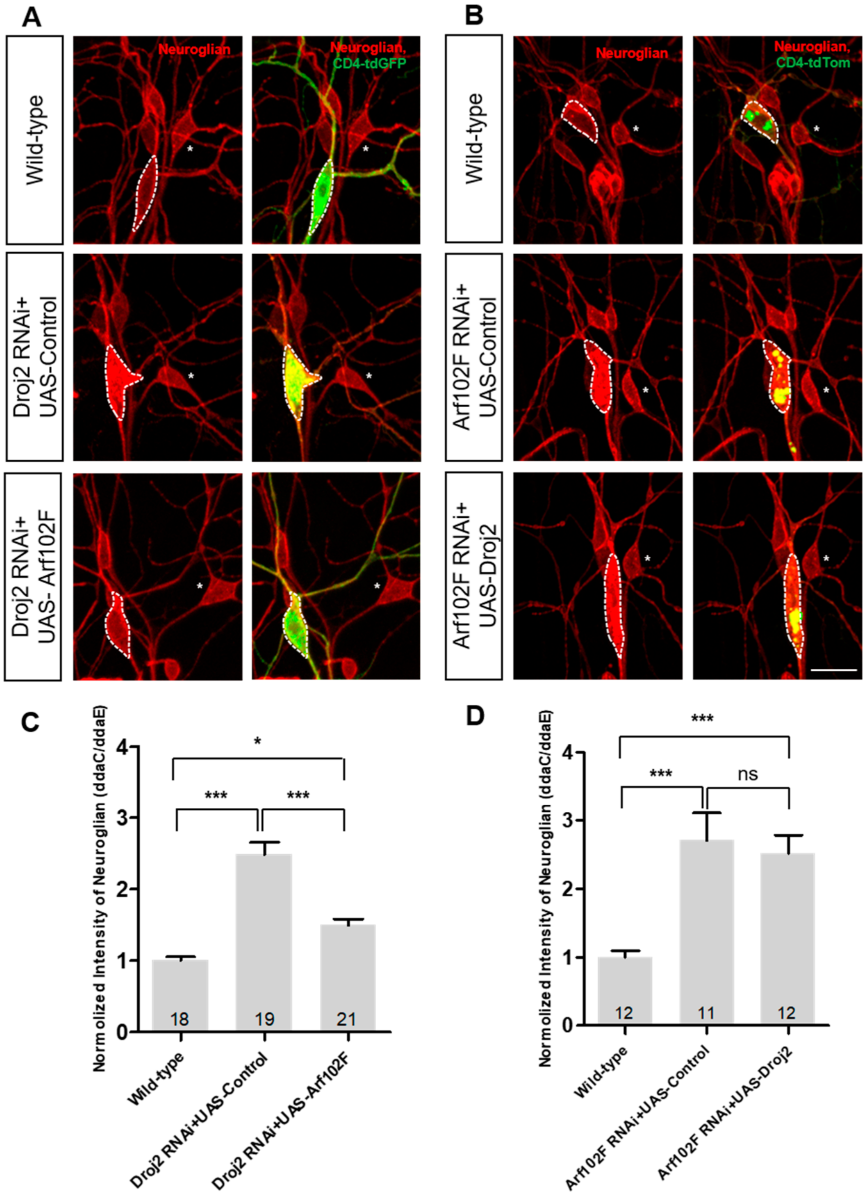

Figure S6. Arf102F Overexpression Partially Alleviates the Neuroglian Defect in Droj2 Downregulated Neurons. (A) Confocal images of wild-type, *droj2* RNAi + UAS–Control, and *droj2* RNAi + UAS–Arf102F C4da neurons that were immunostained for Neuroglian at the WP stage. (B) Confocal images of wild-type, *arf102*f RNAi + UAS–Control, and *arf102*f RNAi + UAS–Droj2 C4da neurons that were immunostained for Neuroglian at the WP stage. ddaC somas are outlined with dashed lines, and ddaE somas are indicated with asterisks. (C–D) Quantitative analysis of normalized Neuroglian fluorescence intensities of the indicated genotypes in C4da somas at the WP stage. In C and D, data are mean±s.e.m. One-way ANOVA with Bonferroni's test was applied to determine statistical significance. ns, not significant; *$P<0.05$; ***$P<0.001$. The number of neurons (n) examined in each group is shown on the bars. Scale bar: 20 μm.
